# Editorial: Polymer Solar Cells: Molecular Design and Microstructure Control

**DOI:** 10.3389/fchem.2020.00697

**Published:** 2020-10-06

**Authors:** Jiangang Liu, Ergang Wang, Kui Zhao

**Affiliations:** ^1^School of Electronics and Information, Northwestern Polytechnical University, Xi'an, China; ^2^Department of Chemistry and Chemical Engineering, Chalmers University of Technology, Gothenburg, Sweden; ^3^School of Materials Science and Engineering, Shaanxi Normal University, Xi'an, China

**Keywords:** solar cells, synthesis, microstructure, morphology, efficiency

Photovoltaic (PV) devices can directly convert sunlight into electricity, which enables a practical and facile solution to address the challenge of the ever-increasing energy demand in a sustainable way. Intensive research and development are searching for high efficiency solar cells with low-cost fabrication. So far PV devices based on various inorganic materials dominate the entire market, including silicon (Si), III-V group semiconductors, CIGS, and CdTe. However, partially due to the related environmental issues and high production cost, traditional PV technologies raise the obvious constraints on the further manufacturing capacity of system-cost, scale-up, and their wide adoption. Recently there has been an ever-growing interest in emerging polymer-based PV technology owing to synthetic variability and low-temperature solution-processing of organic semiconductors, and the capacity of lightweight, flexible, easy and low-cost manufacturing of devices. It is our great pleasure to propose this special issue entitled “Polymer Solar Cells: Molecular Design and Microstructure Control” for *Frontiers in Chemistry*. The issue highlights important aspects of structure-function relationship from both molecular design and microstructure control perspectives. We present a collection of 11 featured articles from this exciting field that covers molecular design of novel materials and microstructure control of the bulk heterojunction (BHJ) layer for high-performance polymer solar cells.

The microstructure of the active layer plays a critical role in photovoltaic outcome. It has been widely believed that percolation networks of both donor and acceptor enable efficient charge transport and collection. Yan et al. found that the weight average molecular weight (M_W_) of polymer had a profound influence on the formation of the percolation network. When the M_W_ of N2200 is larger than 96 kDa, a percolation network structure is formed in the J51:N2200 blend film due to the chain tanglement and multi-chain aggregations, resulting in increased electron mobility and improved device performance (Yan et al.).

Domain sizes should be carefully controlled owing to exciton diffusion length of only 10–20 nm. While it still remains challenging to control the domain sizes as the film formation is more related to thermodynamic process, inhibiting the crystallization of the donor and/or acceptor could reduce the domain size effectively. Liu J. et al. synthesized random copolymers PNDI-Px by introducing porphyrin unit into NDI-based polymer. Due to the more flexible mainchain, the crystallinity of PNDI-Px was suppressed, achieving a finer phase separation structure (Liu J. et al.). In addition, Huang et al. developed a new strategy to control the polymer regiochemistry, which may be used as an effective method to control the crystallinity of polymers and further regulate the phase separation degree. Small molecules are often characterized by high crystallinity. Liu C. et al. synthesized a new small molecule donor with an acceptor-donor-acceptor structure, namely DRTB16-FT, having fluorine atoms on the thienyl substituent of the central benzodithiophene unit. It shows a low-lying HOMO energy level of −5.64 eV, which is beneficial for reducing energy loss when blending with an acceptor. However, the strong aggregation of DRTB16-FT induced the formation of large domain size, causing a low device performance (Liu C. et al.). Li et al. demonstrated that a propeller-like structure could prevent the small molecules from aggregating into undesired large crystals. They designed and synthesized three new propeller-like PDI derivatives with all-fused rigid structures, which showed a more suitable absorption range and charge transport abilities than that of unfused counterparts. Using PTB7-Th as donor and propeller-like PDI derivative as acceptor, micrometer-sized crystals which have been widely found in conventional PDI based solar cells were efficiently diminished (Li et al.).

The vertical phase separation also plays a crucial role in charge transport along the perpendicular direction. In an optimized vertical phase separation, the donor should be enriched at the anode and the acceptor should accumulate at the cathode. Shao et al. systematically examine how fluorine substituent impacts vertical phase separation. They found that the fluorination of a polymer enables better vertical phase separation in the blend film, which facilitates more efficient charge generation and extraction (Shao et al.). Nowadays the vertical phase separation is measured by non-*in-situ* methods, which exhibit high cost, complicated operation and low precision. Feng et al. proposed an *in-situ* measurement method in combination with a self-developed *in-situ* instrument. This diagnostic method is easily accessible and equipped in laboratories, which provides a convenient way to investigate the film-depth-dependent optical and electronic properties (Feng et al.).

The use of a ternary active layer, which is fabricated by introducing a second donor or acceptor into a binary D:A blend is emerging as a promising strategy to improve the device performance. The third component could not only broaden the absorption bandwidth but also regulate the morphology of the active layer. Zhang et al. introduced trifluoromethyl on a newly synthesized small molecular DTBO to strengthen hydrogen bonding between DTBO and the acceptor. The hydrogen bonding has a strong impact on electrostatic potential and benefits π-π stacking in the active layer, leading to superior charge extraction and low charge recombination (Zhang et al.). The addition of a third component also increased the hole mobility of active layer. Liu X. et al. incorporated a state-of-the-art narrow bandgap polymer (PTB7-Th) into the PBDT-TAZ:NOE10 binary system, which enhanced the photovoltaic performance and thermal stability of the device. The improved photovoltaic performance partly benefits from the improved hole mobility, resulting in more efficient charge generation, and balanced charge transport (Liu X. et al.). The photophysical processes of ternary solar cells are investigated by Yin et al. through introducing a small molecule donor BTR as a third component to the PCE-10:PC71BM binary system. It was shown that photocarrier losses via recombination are mitigated and the voltage losses are slightly suppressed in the ternary system, leading to improved performance (Li et al.).

This special issue thus represents the state-of-the-art in this challenging and fascinating scientific area. We are greatly indebted to all authors for their significant contributions and enthusiastic support. We also thank *Frontiers in Chemistry* for their great editorial support. We sincerely hope that this special issue can contribute to better understanding of the fundamental structure-function relationship in organic PV, and that the readers of *Frontiers in Chemistry* will find this special issue helpful and enjoy it!

## Author Contributions

JL collected the manuscript and was in charge of reviewing manuscript. KZ organized this issue and was in charge of reviewing manuscript. EW was in charge of reviewing manuscript. All authors contributed to the article and approved the submitted version.

## Conflict of Interest

The authors declare that the research was conducted in the absence of any commercial or financial relationships that could be construed as a potential conflict of interest.

